# Negative Ion Mode Collision-Induced Dissociation for Analysis of Protein Arginine Methylation

**DOI:** 10.1007/s13361-019-02176-9

**Published:** 2019-03-26

**Authors:** Ksenia Katsanovskaja, Taran Driver, Rüdiger Pipkorn, Marina Edelson-Averbukh

**Affiliations:** 1 Department of Chemistry, Imperial College London, London, SW7 2AZ UK; 2 Department of Physics, Imperial College London, London, SW7 2AZ UK; 3 Department of Translational Immunology, German Cancer Research Centre, INF 580, 69120 Heidelberg, Germany

**Keywords:** Arginine methylation, Collision-induced dissociation, HCD, Negative ion mode MS/MS

## Abstract

Arginine methylation is a common protein post-translational modification (PTM) that plays a key role in eukaryotic cells. Three distinct types of this modification are found in mammals: asymmetric N^η1^N^η1^-dimethylarginine (aDMA), symmetric N^η1^N^η2^-dimethylarginine (sDMA), and an intermediate N^η1^-monomethylarginine (MMA). Elucidation of regulatory mechanisms of arginine methylation in living organisms requires precise information on both the type of the modified residues and their location inside the protein amino acid sequences. Despite mass spectrometry (MS) being the method of choice for analysis of multiple protein PTMs, unambiguous characterization of protein arginine methylation may not be always straightforward. Indeed, frequent internal basic residues of Arg methylated tryptic peptides hamper their sequencing under positive ion mode collision-induced dissociation (CID), the standardly used tandem mass spectrometry method, while the relative stability of the aDMA and sDMA side chains under alternative non-ergodic electron-based fragmentation techniques, electron-capture and electron transfer dissociations (ECD and ETD), may impede differentiation between the isobaric residues. Here, for the first time, we demonstrate the potential of the negative ion mode collision-induced dissociation MS for analysis of protein arginine methylation and present data revealing that the negative polarity approach can deliver both an unambiguous identification of the arginine methylation type and extensive information on the modified peptide sequences.

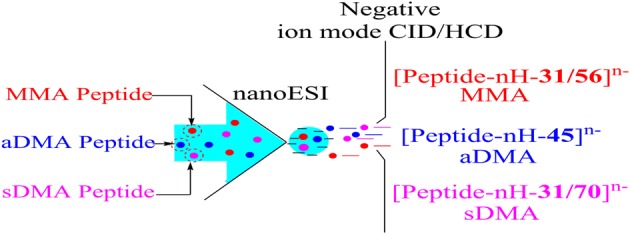

## Introduction

**Figure 1:**
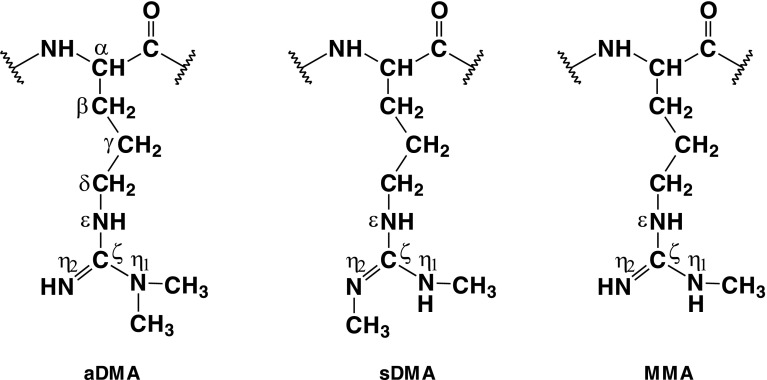
N^η^-methylarginine residue types: aDMA- asymmetric N^η1^, N^η1^-dimethylarginine; sDMA-symmetric N^η1^, N^η2^-dimethylarginine; MMA- N^η1^-monomethylarginine

Protein arginine methylation is a widespread post-translational modification (PTM) that is implicated in a variety of central cellular processes, such as transcription, signal transduction, DNA repair, and apoptosis [1]. This covalent modification has been found to be associated with multiple diseases including cancer, metabolic and cardiovascular disorders, and HIV [2–4]. In humans, arginine methylation is catalyzed by enzymes called protein arginine methyltransferases (types I and II) that give rise to asymmetric N^η1^, N^η1^- and symmetric N^η1^, N^η2^-dimethylated arginines (aDMA and sDMA, respectively) as well as to the intermediate form of the modified residue, N^η1^-monomethylarginine (MMA) [5](see Figure 1). Arginine methylation most frequently occurs in peptide/protein amino acid sequence regions enriched with glycine and arginine residues, denoted as glycine arginine rich (GAR) or RGG motifs [6].

Multiple arginine (Arg) methylation sites have been identified in peptides and proteins to date [7, 8]. The identification of many of these, however, is based on protein sequence similarities (e.g., the presence of the GAR motifs) leaving uncertainty in the identification of the exact modification sites and in the characterization of the responsible methyltransferases [9]. Protein Arg methylation analysis is challenged by the modification cell cycle-dependent character, frequently low stoichiometries and by limitations of the currently available analytical tools [10, 11]. For many years, common ways to analyze these post-translational modifications (PTMs) include the application of classical biochemical methods such as Edman sequencing and amino acid analysis [12, 13], in vitro [^3^H]methyl incorporation combined with fluorography of separated proteins [14], and western blotting with methylarginine-specific antibodies [15] as well as others. These approaches frequently turn out to be highly laborious, may require large amounts of sample, and may suffer from limited specificity for producing conclusive results [16].

In recent decades, mass spectrometry (MS) has become a method of choice for analysis of protein PTMs, owing to its sensitivity, flexibility, and speed [17]. In a typical MS-based proteomic experiment, the proteins are first digested into a mixture of peptides which are subsequently protonated and subjected to a tandem mass spectrometry (MS/MS) analysis, which is followed by database search or de novo sequencing to identify the amino acid sequence of the original proteins and their possible PTMs [17]. In the case of arginine methylation, the MS-based analysis may present a challenge. Indeed, multiple peptides can display the + 14.016 Da and + 28.031 Da mass shifts that mimic an addition of one or two methyl groups to Arg, but in fact carry the modifications of different amino acid residues. For example, the 14/28 Da mass gain can result from methylation of lysine, Gln → Arg amino acid substitution, methylation of glutamic or aspartic acids induced by sample preparation artifacts, and others [6, 18]. False positive Arg methylation assignments may be generated in cases when the alternative peptide modification sites are located in the vicinity of the peptide arginine residues. Since the widespread endoprotease trypsin has poor activity at the alkylated Arg sites [19, 20], many arginines methylated tryptic peptides also contain internal basic residues that cause suppression of the peptide sequencing using collision-induced dissociation (CID) [21, 22].

Arginine methylation marker ions (PTM specific fragment ions of the modified peptides) have been reported to greatly facilitate the positive ion mode mass spectral analysis of this covalent modification [23–26]. Thus, under quadrupole ion trap CID, protonated sDMA-containing sequences undergo characteristic eliminations of monomethylamine (MW = 31 Da) and dimethylcarbodiimide (70 Da) that enable one to distinguish them from the asymmetrically modified aDMA peptide analogues that instead expel dimethylamine (MW = 45 Da) and dimethylguanidine (87 Da) neutrals [27, 28]. Similarly, protonated MMA-containing sequences were shown to lose monomethylguanidine (73 Da) and monomethylcarbodiimide (56 Da) molecules during MS/MS [18, 29–31]. Several low-mass marker ions were also reported for the DMA-peptides [30–32]. However, the formation efficiency of the positive ion mode Arg methylation marker ions has been found to be dependent on multiple factors, such as specific amino acid sequence, number of modified residues within analyzed peptide, peptide charge state, instrumentation type [30, 31, 33], which may cause uncertainty in the practical use of the PTM-specific fragments for the MS analysis.

The alternative common electron-based tandem mass spectrometry methods, such as ETD and ECD [34, 35], in many cases enable one to overcome these limitations. For example, despite the fact that ETD fragmentation is less susceptible to side-chain cleavages compared with CID, the MMA- and DMA-specific neutral losses have been reported for arginine methylated peptides under these conditions [36]. As a result, both an efficient sequencing and determination of Arg methylation type become available through ETD for a multitude of the modified peptides. For example, a targeted data acquisition approach for *Saccharomyces cerevisiae* Npl3 protein digest mixtures has demonstrated that ETD outperforms CID across all observed protonated methylated peptide charge states and masses [37]. Nevertheless, the efficiency of the side chain fragmentation of Arg methylation-containing peptides under ETD is affected by the peptide amino acid sequence [37]. As a result, the PTM-specific decomposition of the methylated peptides may turn out to be suppressed [29] creating a challenge for determination of the arginine methylation type.

Despite the fact that positive ion mode–based mass spectrometry is used standardly for structural analysis of proteins, its ionization bias is well established. Indeed, peptide and protein protonation efficiency under both electrospray and matrix-assisted laser-induced dissociation ionization (ESI and MALDI, respectively) is strongly dependent on the amino acid content and the sequence of the analyzed compounds [38–40]. It is reasonable to assume that MS-based analysis of protein PTMs combining both positive and negative polarities could enhance considerably the robustness of the experimental data in comparison with that of generated through the exclusive use of the positive ion mode approach. So far, only a very limited number of protein PTMs (phosphorylation, sulfation, intramolecular disulfide bridging, and several more [23, 24, 41–46]) can be examined using negative polarity MS/MS, because of the lack of knowledge available on decomposition channels of the corresponding negatively charged modified peptides. Here, for the first time, we report the negative ion mode CID chemistry of Arg methylated peptides demonstrating the potential of the negative polarity MS/MS for the analysis of protein arginine methylation. In this study, we employ a variety of synthetic Arg methylated peptides, including those with the modified Arg/Gly motifs, and demonstrate that the deprotonated modified species undergo both PTM-specific side chain cleavages and extensive backbone dissociation under CID and higher energy collision-induced dissociation (HCD). The negative ion mode MS/MS enables one to identify the type of Arg methylation along with its location within the analyzed peptide sequences.

## Experimental

### Mass Spectrometry

Nanoelectrospray (nanoESI)-MS and MS/MS spectra of peptides were acquired on an LTQ Orbitrap mass spectrometer (Thermo Fisher Scientific, Bremen, Germany) equipped with a robotic nanoflow ion source NanoMate HD System (Advion Biosciences, Inc., Ithaca, NY, USA). Intact peptide ions were detected by the Orbitrap mass analyzer at a mass resolution of 60,000 (at *m/z* 400) and subjected to tandem MS analysis. Two MS/MS techniques were used: CID taking place in a linear ion trap (IT-Orbitrap CID) and the higher energy dissociation (HCD) in a dedicated octopole collision cell installed at the far end of the C-trap of the mass spectrometer [47]. In the HCD experiments, the fragmented ions were transferred back through the C-trap for analysis by the Orbitrap mass analyzer. Both ion trap CID and the HCD tandem mass spectra were acquired with Orbitrap target mass resolution of 7500 (at *m/z* 400). Normalized collision energies (NCE) between 0 and 30% were applied for the IT-Orbitrap CID measurements, and collision energies (CE) of 20–85 eV were used for the HCD experiments. A *q*-value of 0.25 and an activation time of 30 ms were applied in IT-Orbitrap CID. An automated gain control (AGC) value of 3 × 10^4^ at a maximum ion accumulation time of 250 ms and precursor isolation window of 1 amu were applied. Peptides were dissolved in a 50%/0.5%/49.5% (vol/vol/vol) mixture of methanol/formic acid/water to a concentration of ~ 10–50 pmol/μL.

### Peptide Synthesis

Analyzed synthetic peptides **1–9** are listed in Table 1. The peptides were prepared by Fmoc strategy [51, 52] for solid-phase synthesis on a multiple automated synthesizers (Syro II, Multisyntech). The peptide synthesis was carried out on preloaded Wang resins. Peptide chain assembly was performed by in situ activation of amino acid building blocks by 2-(1*H*-benzotriazole-1-yl)-1,1,3,3-tetramethyluronium hexafluorophosphate. The used unmodified amino acids and the modified building blocks Fmoc-Arg-(Me)-OH, Fmoc-Arg-(sMe_2_)-OH, and Fmoc-Arg-(aMe_2_)-OH were purchased from Bachem AG. The synthesized peptides were purified by preparative HPLC on a Kromasil 100–10C 10 μm 120 Å reverse phase column (20 **×** 150 mm) using an eluent of 0.1% trifluoroacetic acid in water (A) and 80% acetonitrile in water (B). The peptides were eluted with a successive linear gradient of 25 to 80% of solvent B for 30 min at a flow rate of 10 ml/min and lyophilized. The purified peptides were characterized by analytical HPLC and MS (Thermo Finnigan, LCQ).

**Table 1:** Model Peptides Used in the Study

Peptide^a^	Molecular weight^c^, Da
TW-**R(*s*CH**_**3**_**)**_**2**_-GGEEK (**1**)	989.4930
TW-**R(*a*CH**_**3**_**)**_**2**_-GGEEK (**2**)	989.4930
TW-R-GGEEK (**3**)	961.4617
G-**R(*s*CH**_**3**_**)**_**2**_-GRPR (**4**) [48, 49]^b^	725.4408
G-**R(*a*CH**_**3**_**)**_**2**_-GRPR (**5**) [48, 49]^b^	725.4408
G-**R(CH**_**3**_**)**-GRPR (**6**) [48, 49]^b^	711.4252
GGNFSG-**R(CH**_**3**_**)**-GGFGGSR (**7**)^d^	1325.6225
GWG-**R(*s*CH**_**3**_**)**_**2**_-EENLFSWK (**8**) [50]^b^	1535.7521
GM-**R(*s*CH**_**3**_**)**_**2**_-G-**R(*s*CH**_**3**_**)**_**2**_-GR (**9**) [50]^b^	844.4813

^a^Arg methylation sites are in bold; R(aCH_3_)_2_ is aDMA; R(sCH_3_)_2_ is sDMA; R(CH_3_) is MMA

^b^Works which reported the modification, these peptide sequences were derived by in-silico digestion of the modified proteins

^c^Peptide monoisotopic molecular weights (in Daltons)

^d^Peptide derived from P09651-ROA1_HUMAN protein (UniProt)

## Results and Discussion

### Arginine Methylation Marker Ions of Deprotonated Peptides

**Figure 2:**
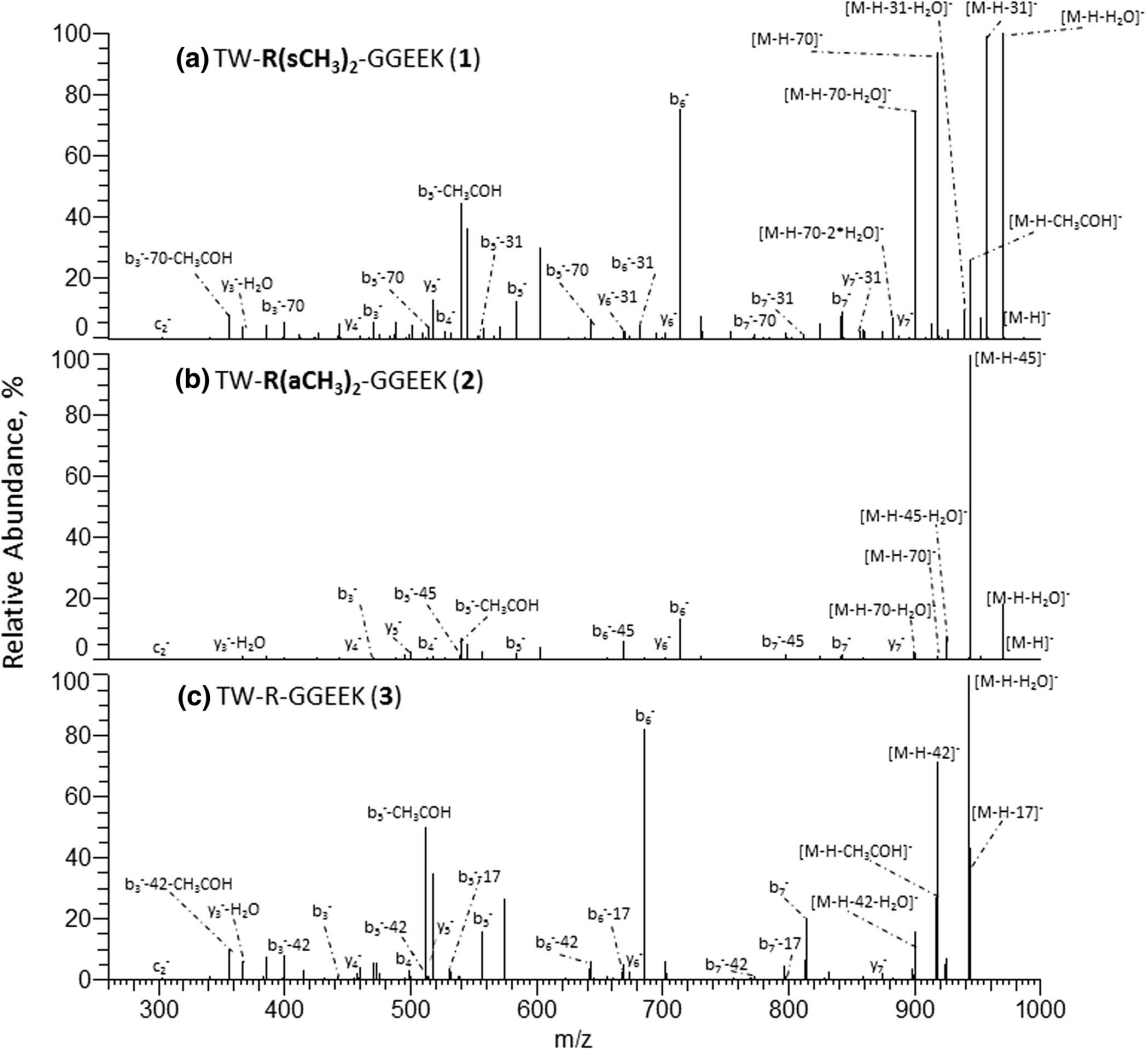
Negative ion mode IT-Orbitrap CID spectra of singly deprotonated Arg dimethylated and unmodified forms (**1**–**3**) of TWRGGEEK peptide. (**a**) CID spectrum of [M-H]^−^ ion of **1**, (**b**) CID spectrum of [M-H]^−^ ion of **2**, and (**c**) CID spectrum of [M-H]^−^ ion of **3**

**Scheme 1:**
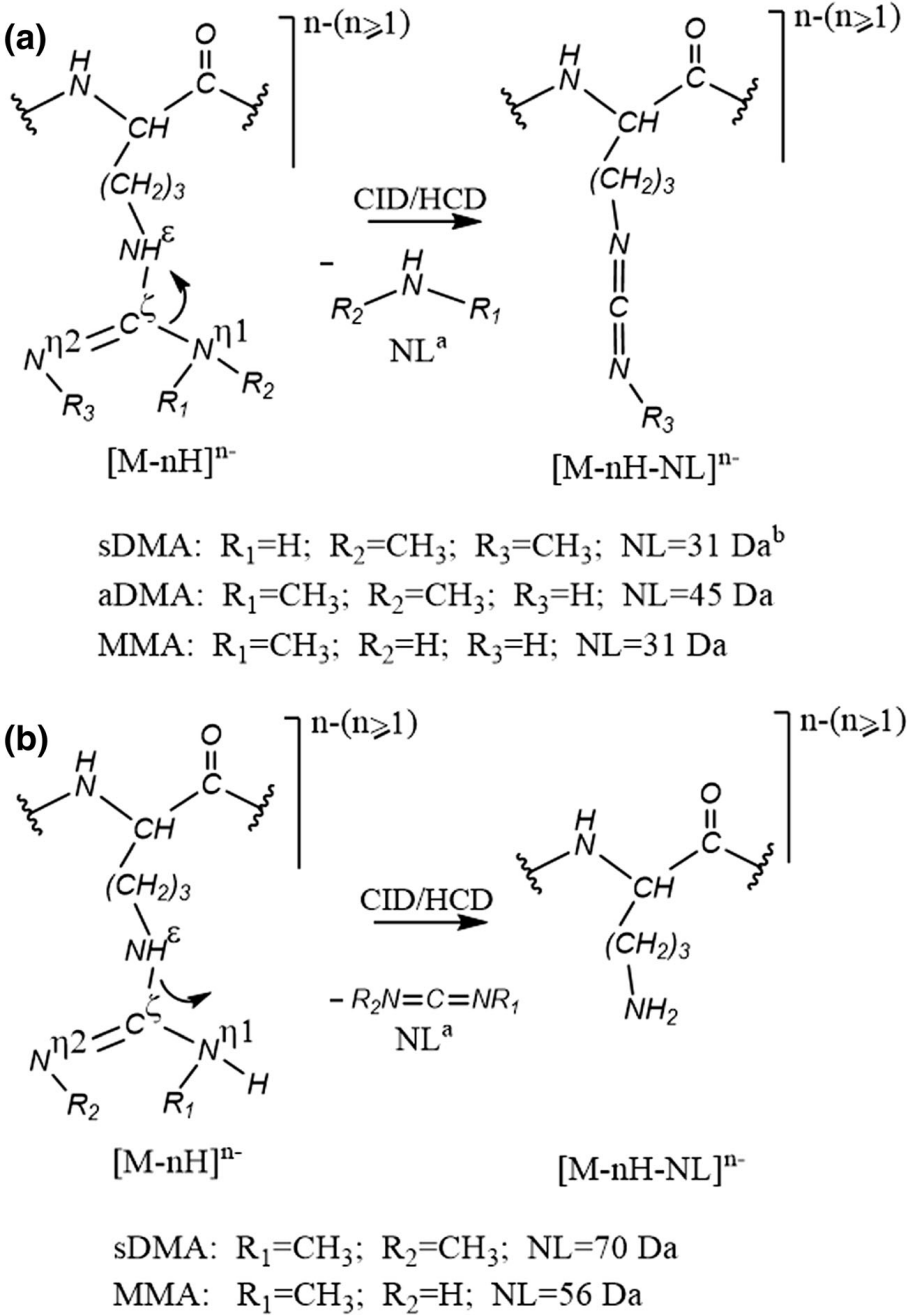
PTM specific side chain dissociation of di- and monomethylarginine-containing peptides under negative ion mode CID. ^a^NL is neutral loss. ^b^Molecular weights of the PTM-specific neutral losses

**Figure 3:**
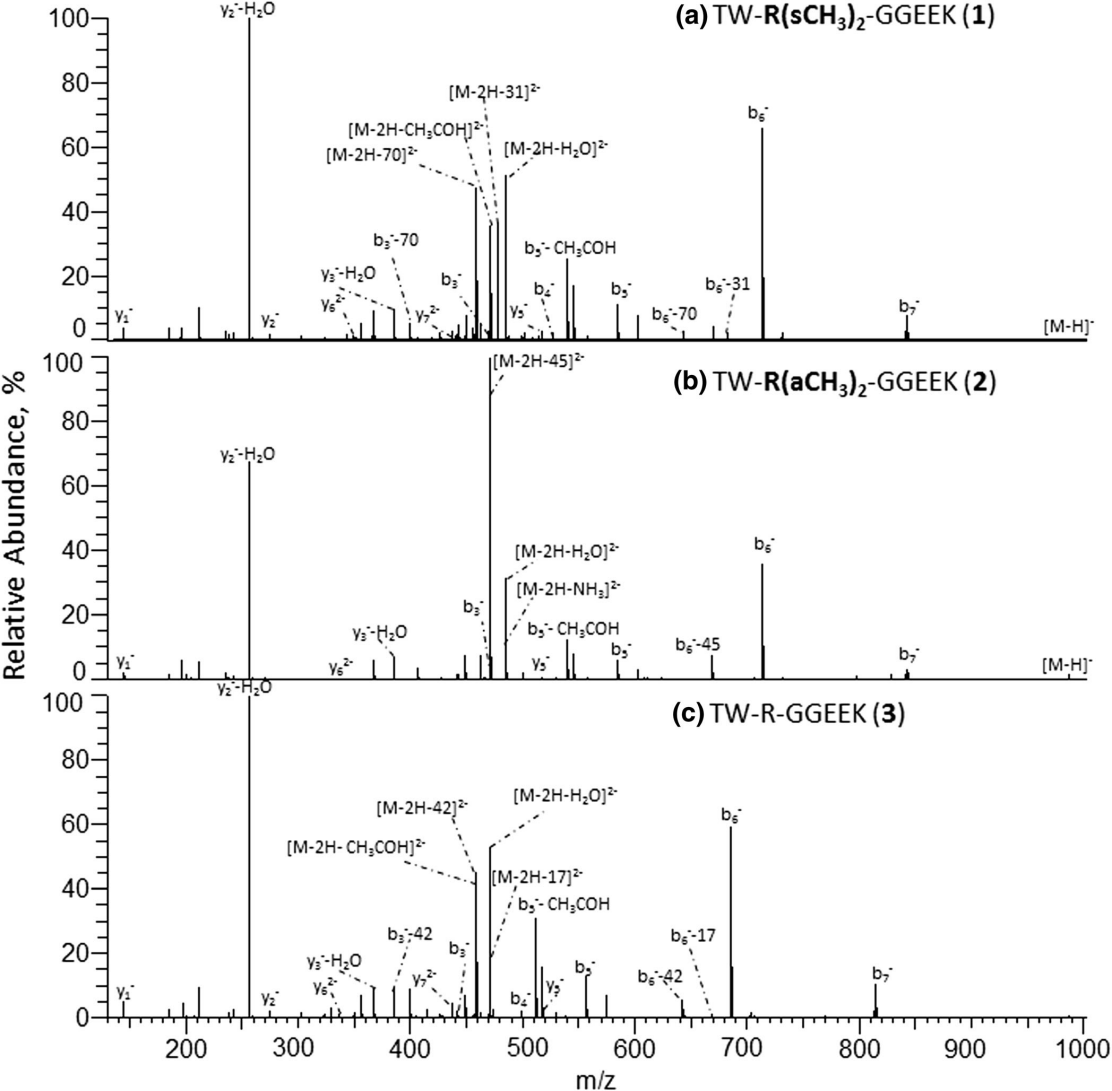
Negative ion mode IT-Orbitrap CID spectra of doubly deprotonated Arg dimethylated and unmodified forms of TWRGGEEK peptide. (**a**) CID spectrum of [M-2H]^2−^ ions of **1**, (**b**) CID spectrum of [M-2H]^2−^ ions of **2**, and (**c**) CID spectrum of [M-2H]^2−^ ion of **3**

NanoESI IT-Orbitrap CID mass spectra of the singly deprotonated isomeric sDMA- and aDMA-peptides **1** and **2** (Figure 2) exhibit a considerable difference. Indeed, the symmetrically dimethylated peptide **1** shows an extensive release of 31 Da and 70 Da neutrals from the parent ion, alone or in combination with other small molecules (Figure 2a), while the CID spectrum of its asymmetrical analogue **2** is dominated by the elimination of a 45 Da neutral molecule (Figure 2b). A comparison of the modified peptide mass spectral data to that of the unmodified analogue **3** (see Figure 2c) reveals that the observed neutral loss reactions are induced by the presence of the modified Arg residues. Indeed, peptide **3** does not expel 31 Da, 45 Da, or 70 Da neutrals under the negative ion mode CID but rather undergoes an extensive elimination of ammonia (17 Da) and carbodiimide (42 Da). Determination of the exact masses of the neutral molecules eliminating from the peptides **1** and **2** reveals that the modified residues of the deprotonated peptides undergo neutral loss of methylamine (31.0422 Da) and dimethylcarbodiimide (70.0531 Da) in the case of the sDMA-peptide **1** and dimethylamine (45.0578 Da) in the case of the aDMA-peptide **2**. A plausible mechanism of the DMA side chain dissociation, involving the modified guanidine C^ξ^–N^η1^ and N^ε^–C^ξ^ bond cleavages off to give rise to the elimination of mono- and dimethylamines (31 Da CH_3_NH_2_ and 45 Da CH_3_NHCH_3_) and dimethylcarbodiimide (70 Da CH_3_N=C=NCH_3_), respectively, is shown in Scheme 1. MS/MS spectra of the neutral loss peptide products provide further confirmation for the DMA-induced origin of the observed decompositions. The abundant DMA marker ions were also observed for the singly and doubly deprotonated molecules of the examined dimethylarginine-containing peptides (**4**, **5**, **8**, **9**), indicating that the 31/45/70 Da neutral losses are characteristic for DMA-peptide negative ion mode CID behavior (see Figure 3, Table 2). Noteworthy, the doubly modified deprotonated peptide **9** undergoes consecutive eliminations of two identical/mixed sDMA-specific neutrals, [M-H-2×31]^−^, [M-H-2×70]^−^, and [M-H-31-70]^−^ (Table 2), which enable one to rapidly recognize the presence of its two sDMA-residues. Analogously, the negative ion mode CID spectra of N^η1^-monomethylated sequences **6** and **7** exhibited a highly efficient release of monomethylated molecules of ammonia (31.0422 Da) and carbodiimide (56.0375 Da) that can be used the PTM marker ions,(see Table 2 and Scheme 1). It is important to point out that the examined deprotonated modified peptides **1**, **2**, **4**–**9** did not show elimination of di- or monomethylated guanidines (87 Da and 73 Da, respectively) under CID that was previously observed in the positive polarity [27].

**Table 2:** Arg-methylation^a^ Specific Neutral Losses of the Modified Peptides Under CID^b^

Peptide^a^	Arg methylation induced neutral loss fragment, RA%	
	[M-H]^−^	[M-2H]^2−^
TW-**R(*s*CH**_**3**_**)**_**2**_-GGEEK (**1**)	[M-H-**31**]^−^ (97%), [M-H-**31**-H_2_O]^−^ (9.4%), [M-H-**70**]^−^ (93.9%), [M-H-**70**-H_2_O]^−^ (73%), [M-H-**70**-2 × H_2_O]^−^ (6.4%), [M-2H-**70**-CH_3_CHO]^2−^ (7.6%)	[M-2H-**31**]^2−^ (35%), [M-2H-**31**-H_2_O]^2−^ (2.7%), [M-2H-**70**]^2−^ (46.5%), [M-2H-**70**-H_2_O]^2−^ (7.4%)
TW-**R(*a*CH**_**3**_**)**_**2**_-GGEEK (**2**)	[M-H-**45**]^−^ (100%), [M-H-**45**-H_2_O]^−^ (7.5%), [M-H-**70**]^−^ (1.2%)	[M-2H-**45**]^2−^ (100%), [M-2H-**45**-H_2_O]^2−^ (7.9%), [M-2H-**45**-H_3_CHO]^2−^ (7.6%)
G-**R(*s*CH**_**3**_**)**_**2**_-GRPR (**4**)	[M-H-**31**]^−^ (100%), [M-H-**31**-NH_3_]^−^ (9.7%), [M-H-**31**-HNCNH]^−^ (11.1%), [M-H-**70**]^−^ (24.6%), [M-H-**70**-NH_3_]^−^ (7%), [M-H-**70**-HNCNH]^−^ (2.7%)	N/A^c^
G-**R(*a*CH**_**3**_**)**_**2**_-GRPR (**5**)	[M-H-**45**]^−^ (100%), [M-H-**45**-HN_3_]^−^ (4.4%), [M-H-**45**-HNCNH]^−^ (6.2%), [M-H-**70**]^−^ (< 0.1%)	N/A^c^
G-**R(CH**_**3**_**)**-GRPR (**6**)	[M-H-**31**]^−^ (100%), [M-H-**31**-NH_3_]^−^ (9%), [M-H-**31**-HNCNH]^−^ (17.4%), [M-H-**56**]^−^ (61.1%), [M-H-**56**-HNCNH]^−^ (4.6%)	N/A^c^
GGNFSG-**R(CH**_**3**_**)**-GGFGGSR (**7**)	[M-H-**31**]^−^ (46%), [M-H-**31**-CH_2_O]^−^ (6.9%), [M-H-**56**]^−^ (40.7%), [M-H-**56**-CH_2_O]^−^ (5.1%)	N/A^c^
GWG-**R(*s*CH**_**3**_**)**_**2**_-EENLFSWK (**8**)	[M-H-**31**]^−^ (81.5%), [M-H-**31**-H_2_O]^−^ (11.6%), [M-H-**31**-2×H_2_O]^−^ (1.1%), [M-H-**70**]^−^ (48.5%), [M-H-**70**-H_2_O]^−^ (41%), [M-H-**70**-2 × H_2_O]^−^ (5.7%)	[M-2H-**31**]^2−^ (67.8%), [M-2H-**31**-H_2_O]^2−^ (7.3%), [M-2H-**31**-CH_2_O]^2−^ (10.5%), [M-2H-**70**]^2−^ (33.5%), [M-2H-**70**-H_2_O]^2−^ (12.3%), [M-2H-**70**-CH_2_O]^2−^ (5.4%), [M-2H-**31**-2 × H_2_O]^2−^ (0.2%)
GM-**R(*s*CH**_**3**_**)**_**2**_-G-**R(*s*CH**_**3**_**)**_**2**_-GR (**9**)	[M-H-**31**]^−^ (100%), [M-H-**31**-HNCNH]^−^ (8.8%), [M-H-**70**]^−^ (31.6%), [M-H-**70**-HNCNH]^−^ (2.6%), [M-H-**2** × **31**]^−^ (12.3%), [M-H-**2** × **70**]^−^ (0.2%), [M-H-**31**-**70**]^−^ (4.3%), [M-H-**31**-**70**-HNCNH]^−^ (0.2%)	N/A^c^

^a^Arg modification sites are in bold

^b^Normalized collision energy 25–30%

^c^The CID data are not available because of strong suppression of the peptide doubly charged molecular ions under negative ion mode nanoESI-MS

**Figure 4:**
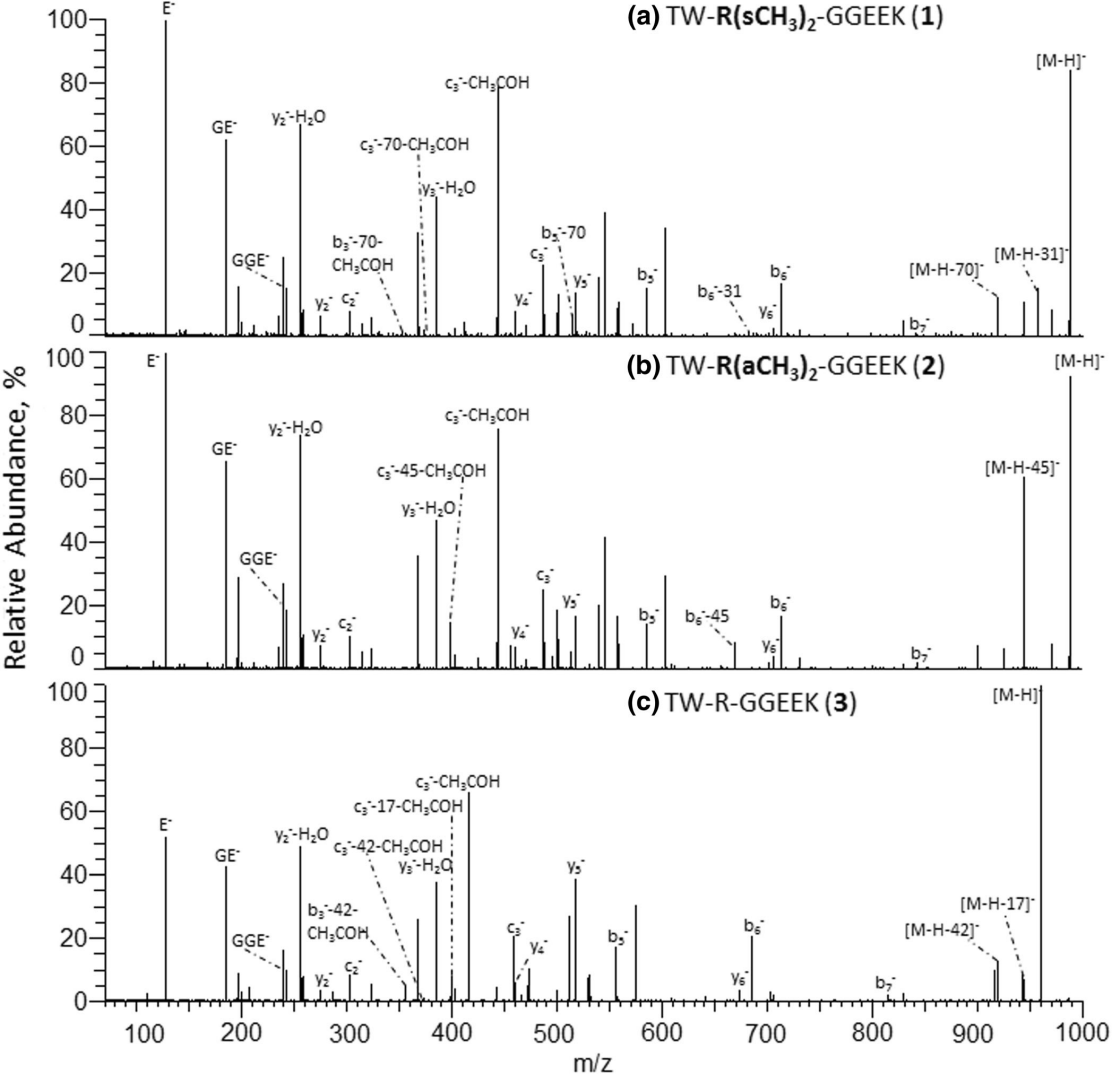
The negative ion mode HCD mass spectra of singly deprotonated Arg dimethylated and unmodified forms of TWRGGEEK peptide. (**a**) HCD spectrum of [M-H]^−^ ion of **1**, (**b**) HCD spectrum of [M-H]^−^ ion of **2**, and (**c**) HCD spectrum of [M-H]^−^ ion of **3**

Energy-resolved negative ion mode HCD experiments were carried out to examine a possible formation of low-mass modification-specific marker fragments during fragmentation of deprotonated Arg methylated peptides that could not be detected under the ion trap CID because of the inherent low-mass spectral cut-off [53]. The HCD arginine methylation-specific reporter fragments of **1**, **2**, **4**–**9** are summarized in Table 3. The experimental data do not reveal an occurrence of the low-mass negatively charged DMA and MMA reporter ions and indicate that HCD arginine methylation marker ions are identical to those observed under the ion trap CID. However, the negative ion mode HCD mass spectra of the peptides **1**, **2**, **4**–**9** display less intense PTM-induced neutral loss signals in comparison with those observed under the IT-Orbitrap CID (see Figures 1 and 4). This observation can be attributed to a more efficient secondary fragmentation inside a collision cell compared with an ion trap [47]. The observed suppression is more pronounced for the peptide [M-2H]^2−^ ions relative to the [M-H]^−^ analogues (see Table 3), as a result of a wider variety of decomposition channels available to the peptide ions carrying more than one charge. Similarly to the ion trap CID, the HCD mass spectra of the modified peptides **1**, **2**, **4**–**9** display the DMA- and MMA-specific neutral loss signals of the corresponding modified backbone fragments (see Figure. 4a and b).

**Table 3:** Characteristic Arg Methylation^a^ Specific Neutral Losses of Peptides **1**–**9** Under HCD^b^

Peptide^a^	Arg methylation induced neutral loss fragment, RA%	
	[M-H]^−^	[M-2H]^2−^
TW-**R(*s*CH**_**3**_**)**_**2**_-GGEEK (**1**)	[M-H-**31**]^−^ (15.5%), [M-H-**31**- CH_3_CHO]^−^ (1.7%), [M-H-**70**]^−^ (12%)	[M-2H-**31**]^2−^ (3.2%), [M-2H-**31**-CH_3_CHO]^2−^ (7.3%), [M-2H-**70**]^2−^ (1.8%), [M-2H-**70**-CH_3_CHO]^2−^ (5.3%)
TW-**R(*a*CH**_**3**_**)**_**2**_-GGEEK (**2**)	[M-H-**45**]^−^ (58.8%), [M-H-**45**- CH_3_CHO]^−^ (8.6%), [M-H-**70**]^−^ (< 0.1%)	[M-2H-**45**]^2−^ (7.6%), [M-2H-**45**-CH_3_CHO]^2−^ (17.2%), [M-2H-**70**]^2−^ (< 0.1%)
G-**R(*s*CH**_**3**_**)**_**2**_-GRPR (**4**)	[M-H-**31**]^−^ (19.1%), [M-H-**31**-NH_3_]^−^ (5.4%), [M-H-**31**-HNCNH]^−^ (7.9%), [M-H-**70**]^−^ (6.2%), [M-H-**31**-NH_3_]^−^ (1.7%)	N/A^c^
G-**R(*a*CH**_**3**_**)**_**2**_-GRPR (**5**)	[M-H-**45**]^−^ (72.8%), [M-H-**45**-NH_3_]^−^ (16.6%), [M-H-**45**-HNCNH]^−^ (31.2%), [M-H-**70**]^−^ (< 0.1%)	N/A^c^
G-**R(CH**_**3**_**)**-GRPR (**6**)	[M-H-**31**]^−^ (27.4%), [M-H-**31**-NH_3_]^−^ (7.9%), [M-H-**31**-HNCNH]^−^ (16.7%), [M-H-**56**]^−^ (18.3%)	N/A^c^
GGNFSG-**R(CH**_**3**_**)**-GGFGGSR (**7**)	[M-H-**31**]^−^ (9.3%), [M-H-**31**-CH_2_O]^−^ (10%), [M-H-**56**]^−^ (17.2%)	N/A^c^
GWG-**R(*s*CH**_**3**_**)**_**2**_-EENLFSWK (**8**)	[M-H-**31**]^−^ (12.6%), [M-H-**31**-H_2_O]^−^ (2.3%), [M-H-**31**-CH_2_O]^−^ (3.3%), [M-H-**70**]^−^ (6.7%)	[M-2H-**31**]^2−^ (2.5%), [M-2H-**70**]^2−^ (0.9%)
GM-**R(*s*CH**_**3**_**)**_**2**_-G-**R(*s*CH**_**3**_**)**_**2**_-GR (**9**)	[M-H-**31**]^−^ (35.8%), [M-H-**70**]^−^ (13.1%), [M-H-**2×31**]^−^ (8.3%), [M-H-**31**-**70**]^−^ (2.1%), [M-H-**2×70**]^−^ (< 0.1%)	N/A^c^

^a^Arg modification sites are in bold

^b^Normalized collision energy 55–65%

^c^The CID data are not available because of a strong suppression of the peptide [M-2H]^2−^ ion formation during negative ion mode nanoESI-MS

**Scheme 2:**
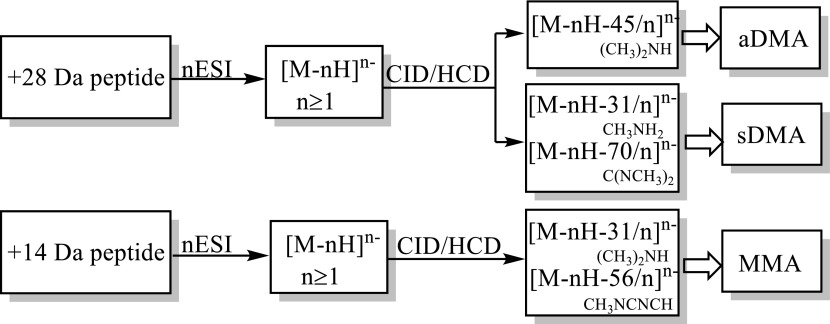
Recognition of DMA and MMA peptide residues using negative ion mode tandem MS data

As a result, the DMA and MMA marker ions of the deprotonated Arg methylated peptides provide a robust means to determine the type of the modified peptide residues under ion trap CID and HCD (see Scheme 2).

### Relative Efficiencies of Peptide Arg Methylation Marker Ion Formation

A comparison of the negative ion mode tandem mass spectra of the isomeric peptides of the **1**, **2** and **4**, and **5** pairs reveals that the sequences carrying asymmetrically dimethylated arginine residues undergo a more efficient PTM-induced dissociation than their sDMA counterparts. Indeed, the relative abundances of the backbone fragments observed in the IT-Orbitrap CID spectra of the deprotonated peptides **2** and **5** are lower than the intensities of the corresponding signals in the mass spectra of the sDMA isomers **1** and **4**, for both singly and doubly charged molecular ions (see Figures 2 and 3). Similarly, under HCD, the RA%s of the peptide aDMA-specific 45 Da loss fragments are considerably higher than those of the 31 Da and 70 Da neutral loss fragments (see Table 3 and Figure 4). The same tendency is observed for the DMA residue-containing backbone fragments (see b_6_^−^/[b_6_-45/31/70]^−^ ion RA% ratios in Figures 2, 3, and 4). The negative ion mode CID and HCD data of the monomethylated peptide **6** reveal that its side chain N^ε^–C^ζ^ bond dissociation is more efficient than that of the sDMA-peptide **4** (see Tables 2 and 3), providing further support for the observed stabilization effect of the N^η1^, N^η2^-arginine dimethylation. A comparison of the Arg methylation marker ion signal intensities of the molecular ion and the backbone fragments of **1** to the corresponding N^ε^–C^ζ^ and C^δ^–N^ε^ bond cleavage products of the unmodified peptide **3** (namely neutral losses of 17 Da NH_3_/42 Da HC=N=CH) demonstrates that the symmetrical methylation of Arg residues facilitates the side chain dissociation relative to the unmodified side chain (see Figures 2, 3, and 4). These experimental data enable us to deduce that the relative decomposition efficiencies of the peptide arginine side chain residues under negative ion mode CID and HCD increase in the following order: Arg < sDMA < MMA < aDMA. The observed effect of the arginine residue methylation on the efficiency of the modified side chain marker ion formation is consistent with N^η^ alkylation-induced guanidine group planarity loss [54] that is especially pronounced in the case of asymmetrically dimethylated arginine, leading to the rapid aDMA-peptide decomposition. It is reasonable to assume that a lower propensity of the N-methylated Arg guanidine group to be involved in a stabilizing intramolecular solvation process within the gaseous modified peptides further contributes to the enhanced dissociation of the alkylated Arg side chains relative to the unmodified analogues.

### Pinpointing the Arg Methylation Sites Under Negative Ion Mode CID and HCD

In addition to the abundant PTM marker ions, the negative ion mode tandem mass spectra of the Arg methylated peptides **1**, **2**, and **4**–**9** show a wide range of sequence-specific backbone fragments. For example, the ion trap CID of the [M-H]^−^ ions of the isomers **1** (sDMA) and **2** (aDMA) produces fragments originated in backbone amide bond cleavages between every pair of the peptide amino acids, enabling one to deduce the full peptide sequences along with unambiguous determination of a type and location of the modified sites (see Figure 2a and b). In this case, indeed, there are four possible potential methylation sites within the isomeric peptides **1** and **2**: Arg-3, Glu-6, Glu-7, and Lys-8. However, the DMA-specific 31 Da/70 Da and 45 Da neutral losses of the deprotonated peptide molecular ions reveal the occurrence of the Arg dimethylation. The position 3 of the modified Arg residues is unambiguously identified based on the 31 Da neutral loss of the dimethylated y_6_^−^ fragment along with the presence of the unmodified y_5_^−^ ion in the case of **1** (Figure 2a), and on the occurrence of the dimethylated b_3_^−^ ion combined with the aDMA-specific neutral losses of the b_5_^−^–b_7_^−^ fragments in the case of **2** (Figure 2b). Noteworthy, despite the fact that the asymmetrically modified peptide **2** produces considerably less abundant backbone fragments in comparison with its symmetrical analogue **1**, its negative ion mode CID data enable one to unambiguously pinpoint the peptide modified residue. Interestingly, the negative ion mode ion trap CID mass spectra of **1** and **2** provide more extensive sequence coverage than their previously reported positive ion mode quadrupole time-of-flight CID analogues [30]. A comparison of IT-Orbitrap CID fragmentation patterns of the [M-H]^−^ ions of **1** and **2** to that of the unmodified analogue **3** (Figure 2) reveals that the Arg dimethylation does not affect the nature of the peptide backbone decompositions.

The ion trap negative ion mode CID mass spectra of [M-H]^−^ ions of **4**–**9** do not provide complete sequence coverage (this varies from 40 to 80%) but enable one to unambiguously pinpoint the modified residue(s) in each of the examined peptides, despite their highly efficient PTM-induced parent ion neutral losses. For example, the correct position of the monomethylated Arg residue within peptide **6**, sequence that contains three arginine residues in total, can be rapidly deduced based on the occurrence of the methylated y_5_^−^ backbone fragment (653.3960) alongside the unmodified forms of the y_2_^−^ and y_4_^−^ ions. The ion trap Orbitrap CID spectra of the [M-2H]^2−^ ions of the modified peptides **1**, **2** and **8** also enable unambiguous pinpointing of the modified residues in each of the cases, with covered sequence fractions varying from 100% for **1** to 71% and 82% for the peptides **2** and **8**, respectively. For example, abundant signals of the [M-2H-31/70 Da]^2−^ and b_4_^−^/[b_4_–70 Da]^−^ fragments in the CID mass spectrum of **8** enable one to identify the symmetrically dimethylated Arg-4 residue. Similarly to the singly charged molecular anions, no effect of the modifying residues on the dianionic peptide backbone decomposition was observed under CID. Our energy resolved negative ion mode IT-Orbitrap CID experiments of the singly and doubly charged molecular ions of the modified peptides **1**, **2**, and **4**–**9** revealed that normalized collisional energies (NCE) of 20–25% can be robustly applied to detect and localize Arg methylation sites for peptides with molecular weights within 700–1500 Da.

Similarly to IT-Orbitrap CID, negative ion mode HCD mass spectra of the modified peptides **1**, **2**, and **4**–**9** enable unambiguous localization of the Arg methylation residues, with amino acid sequence coverage varying between 83 and 100% in the case of molecular monoanions. For the doubly charged molecular species, the HCD sequence coverage appears to be more sequence dependent, reaching 60% for the peptides **1** and **2** and 82% for **8**. Despite the relatively low sequence coverage provided by the HCD data for the doubly charged peptides **1** and **2**, the mass spectra enable the unambiguous localization of the dimethylated arginine residues in both cases (see Figure 4a and b). Indeed, elimination of the 31/70 Da and 45 Da neutrals observed from the [c_3_-CH_3_COH]^−^ fragments of **1** and **2** provides direct evidence for the presence of sDMA/aDMA residues within the three N-terminal amino acids while that of the unmodified c_2_^−^ fragments proves unambiguously the Arg-3 position of the modifications. Collision energies of 55–65 eV were found to be suitable for the arginine methylation analysis of both singly and doubly deprotonated modified peptides **1**, **2**, and **4**–**9** under HCD. It is important to point out that the negative ion mode HCD mass spectra of the analyzed peptides **1**–**9** exhibit more intense y-type fragment signals in comparison with the corresponding b-ions (see [y_2_-H_2_O]^−^ vs b_6_^−^ fragment ion RAs in Figure 4a–c). This is in contrast to the IT-Orbitrap CID behavior of the negatively charged sequences (see Figure 2 where the b_6_^−^ ions produce the most abundant peptide backbone fragment signals). It is reasonable to assume that the observed more efficient y-type ion formation under HCD stems from the negative charge relocalization over the C-terminus of the activated y-type ions to give rise to the resonantly stabilized terminal carboxylate ions that are not available in the case of the b-ions. The HCD mass spectra of the modified and unmodified peptides **1**–**9** also reveal peptide backbone fragments that are not observed in their ion trap CID mass spectra. For the peptides **1**–**3**, for example, these include internal b-ions E^−^, GE^−^, and GGE^−^ that are generated following two backbone cleavages, or the peptide c-type ions [c_3_-CH_3_COH]^−^ produced by the efficient cleavage of the Gly–4N–C_α_ bond. The observed facile elimination of acetaldehyde is originated in the peptide Thr-1 side chain decomposition [42].

### Comparison of Negative and Positive Ion Mode Fragmentation of Arg Methylated Peptides

**Figure 5:**
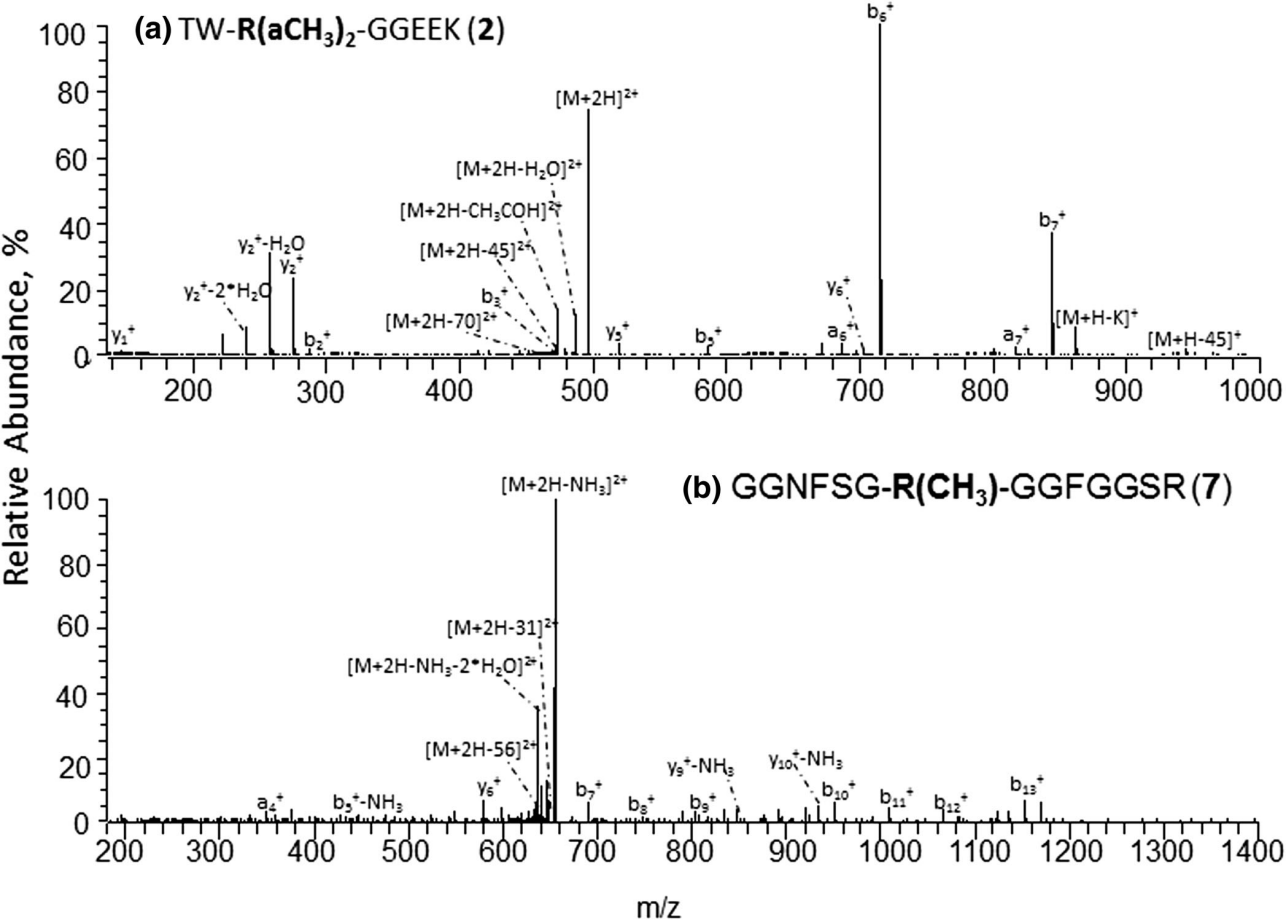
Ion trap Orbitrap CID mass spectra of doubly protonated Arg methylated peptides. (**a**) CID spectrum of [M+2H]^2+^ ion of **2**, (**b**) CID spectrum of [M+2H]^2+^ ion of **7**

The modified peptides **1**, **2**, and **4**–**9** were also analyzed by positive ion mode IT-Orbitrap CID and HCD, in order to compare the structural information available from both polarities. The MMA and DMA reporter ions of the protonated Arg methylated peptides were found to be identical to those observed in the negative ion mode. However, the positive ion mode PTM-induced marker fragments produced much less abundant signals relative to their negative ion mode counterparts, with especially high suppression observed for the aDMA sequences (see the [M+nH-45 Da]^2+^ signal in Figure 5a). Higher charge states of the examined triply protonated modified peptides ([M+3H]^3+^) contributed to further suppression of the peptide Arg methylation marker ions in comparison with the 2+ analogues. The observed aDMA dissociation suppression trend of the protonated Arg methylated peptides is opposite to that of the negatively charged sequences (see data for isomeric peptides pairs **1**, **2** and **4**, and **5** in Tables 2 and 3). This phenomenon can be explained by the fact that in contrast to the negative ion mode PTM marker ion formation for the positively charged Arg methylated peptides requires protonation of their modified residues [31] that interferes with the steric hindrance produced by the guanidine methyl groups of the methylated arginines (particularly strong in the case of aDMA residues [54]). These data suggest that the negative ion mode approach can provide a more reliable recognition of protein Arg methylation type in comparison with the positive ion mode, especially for Arg methylated peptides with additional basic residues that may sequester the external proton causing further suppression of the protonated peptide PTM specific fragmentation.

Interestingly, despite the lower relative abundances of the arginine methylation reporter ions in the positive ion mode IT-Orbitrap CID and HCD, the protonated peptide tandem MS data may generate less sequence information compared with the negative polarity. For example, for the protonated modified peptides **1** and **2**, the CID or HCD spectra of the peptide [M+2H]^2+^ or [M+3H]^3+^ ions do not provide the complete amino acid sequence, in contrast with the negative ion mode analysis (Figure 2a and b). The observed suppression of the protonated modified peptide backbone decomposition under MS/MS is most probably caused by the expected sequestering of the external proton on the analyzed peptide internal basic residues [22]. The experimental data obtained for the Arg methylated peptides **1**, **2**, and **4**–**9** demonstrate that the CID and HCD mass spectra acquired in two different polarities provide on many occasions complementary information regarding the amino acid sequences. Thus, for example, in contrast with the positive ion mode, the relative positions of the two N-terminal residues Thr-Trp in peptides **1** and **2** can be rapidly identified based on the observed y_7_^−^ fragment and its derivatives from the corresponding [M-H]^−^ ions (see Figure 2). From the other side, in the case of the [M+2H]^2+^ ion of sDMA peptide **8**, the CID mass spectrum enables one to identify the relative positions of its Ser-10 and Trp-11 residues (based on the formation of the corresponding y_2_^+^ and b_10_^+^ ions), while the analogous backbone cleavages do not occur in negative ion mode CID or HCD. In contrast with the positive ion CID or HCD, in addition to the canonical b- and y-type fragments, the negative polarity tandem mass spectra of **1**, **2**, and **4**–**9** also display products of amino acid N–C_α_ bond cleavages (e.g., c_2_^−^ ion in Figure 2), resulting in a more diverse array of fragment ions for the analyzed peptides. The observed differences in the fragmentation patterns of the protonated and the negatively charged Arg methylated peptides can be explained by the distinct nature of the gas-phase decompositions in the two polarities. The backbone cleavages of positively charged peptides are initiated by the external proton attachment to the backbone amides [55] while that of the deprotonated sequences is induced by a proton abstraction from the peptide amino acid C_α_ and C_β_ groups [56].

## Conclusions

Our results reveal for the first time that negative ion mode collision-induced dissociation is a powerful tool for analysis of N^η^-methylated arginine residues of peptides and proteins. We demonstrate that negative ion mode CID can be used for both detection and pinpointing of the modified amino acids. We have identified unique marker ions for peptides containing asymmetric N^η1^, N^η1^- and symmetric N^η1^, N^η2^-dimethylarginines as well as N^η1^-monomethylarginine residues under negative ion mode ion trap CID and HCD. In contrast with the positive ion mode CID, fragmentation of the modified peptide anions is not compromised by the peptide internal basic residues, enabling it to provide an extensive sequence information along with the reliable recognition of the arginine methylation types. Our data show that the negative ion mode fragmentation enables one to cover parts of the modified peptide sequences different from those available from the standard positive ion mode CID analysis. Our results suggest that taking advantage of the inherent differences between the gas phase chemistries of the deprotonated and positively charged arginine methylated peptides, e.g., via adapting to a dual polarity approach, may considerably boost the MS analysis of protein arginine methylation sites.

## References

[1] Blanc RS, Richard S (2017). Arginine methylation: the coming of age. Mol. Cell.

[2] Stein C, Riedl S, Rü D, Reiner R, Tzold N, Bauer U-M (2012). The arginine methyltransferase PRMT6 regulates cell proliferation and senescence through transcriptional repression of tumor suppressor genes. Nucleic Acids Res..

[3] Chandrasekharan UM, Wang Z, Wu Y, Wilson Tang WH, Hazen SL, Wang S, Elaine Husni M (2018). Elevated levels of plasma symmetric dimethylarginine and increased arginase activity as potential indicators of cardiovascular comorbidity in rheumatoid arthritis. Arthritis Res. Ther..

[4] Copeland RA, Solomon ME, Richon VM (2009). Protein methyltransferases as a target class for drug discovery. Nat. Rev. Drug Discov..

[5] Gary JD, Clarke S (1998). RNA and protein interactions modulated by protein arginine methylation. Prog. Nucleic Acid Res. Mol. Biol..

[6] Pahlich S, Zakaryan RP, Gehring H (2006). Protein arginine methylation: cellular functions and methods of analysis. Biochim. Biophys. Acta.

[7] Wesche J, Kühn S, Kessler BM, Salton S, Wolf A (2017). Protein arginine methylation: a prominent modification and its demethylation. Cell. Mol. Life Sci..

[8] Wooderchak WL, Zang T, Zhou ZS, Acuña M, Tahara SM, Hevel JM (2008). Substrate profiling of PRMT1 reveals amino acid sequences that extend beyond the “RGG” paradigm. Biochem..

[9] Boisvert F-M, Côté J, Boulanger M-C, Richard SA (2003). Proteomic analysis of arginine-methylated protein complexes. Mol. Cell. Proteomics.

[10] Lakowski TM, Szeitz A, Pak ML, Thomas D, Vhuiyan MI, Kotthaus J, Clement B, Frankel A (2013). MS3 fragmentation patterns of Monomethylarginine species and the quantification of all Methylarginine species in yeast using MRM3. J. Proteome.

[11] Ostareck-Lederer A, Ostareck DH, Rucknagel KP, Schierhorn A, Moritz B, Huttelmaier S, Flach N, Handoko L, Wahle E (2006). Asymmetric arginine dimethylation of heterogeneous nuclear ribonucleoprotein K by protein-arginine methyltransferase 1 inhibits its interaction with c-Src. J. Biol. Chem..

[12] Lin WJ, Gary JD, Yang MC, Clarke S, Herschman HR (1996). The mammalian immediate-early TIS21 protein and the leukemia-associated BTG1 protein interact with a protein-arginine N-methyltransferase. J. Biol. Chem..

[13] Ghosh SK, Paik WK, Kim S (1988). Purification and molecular identification of two protein methylases I from calf brain. Myelin basic protein- and histone-specific enzyme. J. Biol. Chem..

[14] Lee J, Cheng D, Bedford MT, Dickson RC (1990). Techniques in protein methylation. Methods in Molecular Biology.

[15] Strahl BD, Briggs SD, Brame CJ, Caldwell JA, Koh SS, Ma H, Cook RG, Shabanowitz J, Hunt DF, Stallcup MR (2001). Methylation of histone H4 at arginine 3 occurs in vivo and is mediated by the nuclear receptor coactivator PRMT1. Curr. Biol..

[16] Komyod W, Bauer U-M, Heinrich PC, Haan S, Behrmann I (2005). Are STATS arginine-methylated?. J. Biol. Chem..

[17] Aebersold R, Goodlett DR (2001). Mass spectrometry in proteomics. Chem. Rev..

[18] Jung SY, Li Y, Wang Y, Chen Y, Zhao Y, Qin J (2008). Complications in the assignment of 14 and 28 Da mass shift detected by mass spectrometry as in vivo methylation from endogenous proteins. Anal. Chem..

[19] Baldwin GS, Carnegie PR (1971). Specific enzymic methylation of an arginine in the experimental allergic encephalomyelitis protein from human myelin. Science.

[20] Merrill BM, Lopresti MB, Stone KL, Williams KRA (1987). Acid sequence of UP1, an hnRNP-derived single-stranded nucleic acid binding protein from calf thymus. Int. J. Pept. Protein Res..

[21] Doll S, Burlingame AL (2015). Mass spectrometry-based detection and assignment of protein posttranslational modifications. ACS Chem. Biol..

[22] Chi A, Huttenhower C, Geer LY, Coon JJ, Syka JEP, Bai DL, Shabanowitz J, Burke DJ, Troyanskaya OG, Hunt DF (2007). Analysis of phosphorylation sites on proteins from saccharomyces cerevisiae by electron transfer dissociation (ETD) mass spectrometry. Proc. Natl. Acad. Sci. U. S. A..

[23] Hung C-W, Schlosser A, Wei J, Lehmann WD (2007). Collision-induced reporter fragmentations for identification of covalently modified peptides. Anal. Bioanal. Chem..

[24] Steen H, Ku B, Fernandez M, Pandey A, Mann M (2001). Detection of tyrosine phosphorylated peptides by precursor ion scanning quadrupole TOF mass spectrometry in positive ion mode. Anal. Chem..

[25] Huddleston MJ, Annan RS, Bean MF, Carr SA (1993). Selective detection of phosphopeptides in complex mixtures by electrospray liquid chromatography/mass spectrometry. J. Am. Soc. Mass Spectrom..

[26] Shishkova E, Zeng H, Liu F, Kwiecien NW, Hebert AS, Coon JJ, Xu W (2017). Global mapping of CARM1 substrates defines enzyme specificity and substrate recognition. Nat. Commun..

[27] Brame CJ, Moran MF, McBroom-Cerajewski LDB (2004). A mass spectrometry based method for distinguishing between symmetrically and asymmetrically Dimethylated arginine residues. Rapid Commun. Mass Spectrom..

[28] Yagüe J, Vázquez J, López de Castro JAA (2000). Post-translational modification of nuclear proteins, N(G),N(G)-dimethyl-Arg, found in a natural HLA class I peptide ligand. Protein Sci..

[29] Wang H, Straubinger RM, Aletta JM, Cao J, Duan X, Yu H, Qu J (2009). Accurate localization and relative quantification of arginine methylation using nanoflow liquid chromatography coupled to electron transfer dissociation and drbitrap mass spectrometry. J. Am. Soc. Mass Spectrom..

[30] Rappsilber J, Friesen WJ, Paushkin S, Dreyfuss G, Mann M (2003). Detection of arginine dimethylated peptides by parallel precursor ion scanning mass spectrometry in positive ion mode. Anal. Chem..

[31] Gehrig PM, Hunziker PE, Zahariev S, Pongor S (2004). Fragmentation pathways of NG-methylated and unmodified arginine residues in peptides studied by ESI-MS/MS and MALDI-MS. J. Am. Soc. Mass Spectrom..

[32] Couttas TA, Raftery MJ, Bernardini G, Wilkins MR (2008). Immonium ion scanning for the discovery of post-translational modifications and its application to histones. J. Proteome Res..

[33] Lau KW, Hart SR, Lynch JA, Wong SCC, Hubbard SJ, Gaskell SJ (2009). Observations on the detection of B- and Y-type ions in the collisionally activated decomposition spectra of protonated peptides. Rapid Commun. Mass Spectrom..

[34] Zubarev RA, Kelleher NL, Mclafferty FW (1998). Electron capture dissociation of multiply charged protein cations. A nonergodic process. J. Am. Chem. Soc..

[35] Syka JEP, Coon JJ, Schroeder MJ, Shabanowitz J, Hunt DF (2004). Peptide and protein sequence analysis by electron transfer dissociation mass spectrometry. Proc. Natl. Acad. Sci. U. S. A..

[36] Snijders AP, Hung ML, Wilson SA, Dickman MJ (2010). Analysis of arginine and lysine methylation utilizing peptide separations at neutral pH and electron transfer dissociation mass spectrometry. J Am Soc Mass Spectrom..

[37] Hart-Smith G, Low JK, Erce MA, Wilkins MR (2012). Enhanced methylarginine characterization by post-translational modification-specific targeted data acquisition and electron-transfer dissociation mass spectrometry. J Am Soc Mass Spectrom..

[38] Thompson AJ, Hart SR, Franz C, Barnouin K, Ridley A, Cramer R (2003). Characterization of protein phosphorylation by mass spectrometry using immobilized metal ion affinity chromatography with on-resin β-elimination and Michael addition. Anal. Chem..

[39] Woods AS, Wang H-YJ, Jackson SN (2007). Sulfation, the up-and-coming post-translational modification: its role and mechanism in protein–protein interaction. J. Proteome Res..

[40] Haselmann KF, Budnik BA, Kjeldsen F, Nielsen ML, Olsen J, V; Zubarev RA (2002). Fragmentation of polypeptide cations and anions electronic excitation gives informative fragmentation of polypeptide cations and anions. Eur. J. Mass Spectrom.

[41] Edelson-Averbukh M, Pipkorn R, Lehmann WD (2006). Phosphate group-driven fragmentation of multiply charged phosphopeptide anions. Improved recognition of peptides phosphorylated at serine, threonine, or tyrosine by negative ion electrospray tandem mass spectrometry. Anal. Chem..

[42] Edelson-Averbukh M, Pipkorn R, Lehmann WD (2007). Analysis of protein phosphorylation in the regions of consecutive serine/threonine residues by negative ion electrospray collision-induced dissociation. Approach to pinpointing of phosphorylation sites. Anal. Chem..

[43] Edelson-Averbukh M, Shevchenko A, Pipkorn R, Lehmann WD (2011). Discrimination between peptide O-sulfo- and O-phosphotyrosine residues by negative ion mode electrospray tandem mass spectrometry. J. Am. Soc. Mass Spectrom..

[44] Edelson-Averbukh M, Shevchenko A, Diger Pipkorn R, Lehmann WD (2009). Gas-phase intramolecular phosphate shift in phosphotyrosine-containing peptide monoanions. Anal. Chem..

[45] Bilusich D, Bowie JH (2009). Fragmentations of (M-H) − anions of underivatised peptides. Part 2: characteristic cleavages of Ser and Cys and of disulfides and other post-translational modifications, together with some unusual internal processes. Mass Spectrom. Rev..

[46] Wang T, Nha Tran TT, Andreazza HJ, Bilusich D, Brinkworth CS, Bowie JH (2018). Negative ion cleavages of (M-H) − anions of peptides. Part 3. Post-translational modifications. Mass Spectrom. Rev..

[47] Olsen JV, Macek B, Lange O, Makarov A, Horning S, Mann M (2007). Higher-energy C-trap dissociation for peptide modification analysis. Nat. Methods.

[48] Sgarra R, Diana F, Bellarosa C, Dekleva V, Rustighi A, Toller M, Manfioletti G, Giancotti V (2003). During apoptosis of tumor cells HMGA1a protein undergoes methylation: identification of the modification site by mass spectrometry. Biochem..

[49] Zou Y, Wang Y (2005). Tandem mass spectrometry for the examination of the posttranslational modifications of high-mobility group A1 proteins: symmetric and asymmetric dimethylation of Arg25 in HMGA1a protein. Biochem..

[50] Boisvert F-M, Côté J, Boulanger M-C, Cléroux P, Bachand F, Autexier C, Richard S (2002). Symmetrical dimethylarginine methylation is required for the localization of SMN in Cajal bodies and pre-mRNA splicing. J. Cell Biol..

[51] Merrifield RB (1963). Solid phase peptide synthesis. I. The synthesis of a tetrapeptide. J. Am. Chem. Soc..

[52] Carpino LA, Han GY (1972). 9-Fluorenylmethoxycarbonyl amino-protecting group. J. Org. Chem.

[53] March RE (1997). An introduction to quadrupole ion trap mass spectrometry. J. Mass Spectrom..

[54] Raman B, Guarnaccia C, Nadassy K, Zakhariev S, Pintar A, Zanuttin F, Frigyes D, Acatrinei C, Vindigni A, Pongor G (2001). Nomega-arginine dimethylation modulates the interaction between a Gly/Arg-rich peptide from human nucleolin and nucleic acids. Nucleic Acids Res..

[55] Paizs B, Suhai S (2005). Fragmentation pathways of protonated peptides. Mass Spectrom. Rev..

[56] Bowie JH, Brinkworth CS, Dua S (2002). Collision-induced fragmentations of the (M-H)? Parent anions of underivatized peptides: an aid to structure determination and some unusual negative ion cleavages. Mass Spectrom. Rev..

